# Autologous antibody to src-homology 3-domain GRB2-like 1 specifically increases in the sera of patients with low-grade gliomas

**DOI:** 10.1186/1756-9966-31-85

**Published:** 2012-10-11

**Authors:** Tomoo Matsutani, Takaki Hiwasa, Masaki Takiguchi, Takashi Oide, Mitoshi Kunimatsu, Naokatsu Saeki, Yasuo Iwadate

**Affiliations:** 1 Departments of Neurological Surgery, Chiba University, Graduate School of Medicine, 1-8-1, Inohana, Chuo-ku, Chiba 260-8670, Japan; 2 Genetics and Biochemistry, Chiba University, Graduate School of Medicine, 1-8-1, Inohana, Chuo-ku, Chiba, 260-8670, Japan; 3 Diagnostic Pathology, Chiba University, Graduate School of Medicine, 1-8-1, Inohana, Chuo-ku, Chiba, 260-8670, Japan; 4 Department of Biochemistry, Graduate School of Life Science, Nagoya Women’s University, 3-40, Shioji-cho, Mizuho-ku, Nagoya, 467-8610, Japan

**Keywords:** Src, SH3GL1, Autoantibody, Glioma, SEREX

## Abstract

**Background:**

Glioma is the most common primary malignant central nervous system tumor in adult, and is usually not curable in spite of various therapeutic approaches. Clarification of the oncogenic process in its early stage is important for the diagnosis and effective therapy.

**Methods:**

In the present study, we used the serological identification of antigens by recombinant cDNA expression cloning (SEREX) to explore the subtle changes of the protein expression in low-grade glioma. The levels of serum autoantibodies to the SEREX-identified glioma-related antigens were analyzed by ELISA, and the epitope site was identified using deletion mutants and overlap peptide array. Changes in the serum autoantibody levels were examined in the rat glioma model using C6 and 9 L glioma cell lines.

**Results:**

We identified 31 glioma-related antigens by SEREX. Among them, the serum level of autoantibody to src-homology 3-domain GRB2-like 1 (SH3GL1) was significantly higher in patients with low-grade glioma than healthy volunteers or high-grade gliomas. The 10 amino-acids at the C-terminal were identified as the epitope site by the overlap peptide array and the ELISA using deletion mutants. The tissue expression of SH3GL1 protein increased in proportion to glioma progression. The rat glioma models confirmed the increase of anti-SH3GL1 autoantibody level in the early stage and the suppression in the late stage.

**Conclusion:**

SH3GL1 may be involved in the oncogenic process of gliomas and effectively elicit an autologous antibody response in low-grade gliomas. The immunological reaction to SH3GL1 would contribute to the establishment of a novel diagnostic and therapeutic target for gliomas.

## Introduction

Glioma is the most common primary malignant central nervous system (CNS) tumor in adults and arises from neuroepithelial cells, mostly astrocytes or oligodendrocytes. Glioma is divided into 4 grades according to World Health Organization (WHO) histological classification, and the prognosis of glioma is still poor [1,2]. Glioblastoma (GB), WHO grade IV, and anaplastic astrocytoma (AA), WHO grade III, are referred to as high-grade glioma, and the median survival time of patients with AA and GB is 2–3 years and only approximately 1.5 years, respectively [2]. In the cases of WHO grade II tumor, the median survival time of patients with diffuse astrocytoma (WHO grade II) is also limited to approximately 5–7 years [3]. In most cases, patients with glioma present large cerebral lesion at diagnosis, which prevents effective removal without neurological deficits, and the remnant tumors relapse even though receiving post-operative treatments with radiotherapy and chemotherapy [4]. The clarification of the oncogenic process especially in the early stage would contribute to its early diagnosis and to new molecular targets.

Serological identification of antigens by recombinant cDNA expression cloning (SEREX) is one of the powerful tools for finding novel cancer antigens [5] and has been applied on a nationwide basis to target many cancers, including gliblastoma [6-8]. However, the specific and crucial changes in the protein expression in low-grade gliomas have not been identified yet. In contrast, it is well known that activation of the receptor tyrosine kinases such as epidermal growth factor receptor (EGFR) is the most frequent molecular aberration found in high-grade gliomas [9]. The receptor tyrosine kinases make the ras pathway activation through a protein-protein interaction of the adaptor protein called GRB2 with Son of Sevenless (Sos) protein through src-homology 3 (SH3) domain [10,11]. The connection of the adaptor protein and Sos is a key step toward activating the ras-mediated oncogenic pathways in the downstream of receptor tyrosine kinases.

In the present study, the authors applied SEREX to glioma to find SH3-domain GRB2-like 1 (SH3GL1) as a novel glioma-related antigen. The levels of serum autoantibodies to SH3GL1 were significantly higher in patients with low-grade gliomas than in healthy donors by ELISA. In contrast, the serum autoantibody level was significantly depressed in high-grade glioma patients compared with low-grade gliomas patients. We identified the epitope site of SH3GL1 by overlap peptide array and an ELISA using deletion mutants. The rat glioma model using C6 and 9 L glioma cells also showed the increases of the anti-SH3GL1 autoantibody level in the early stage and decreases in the late stage. Although low-grade gliomas are not always in an early-stage of the disease, it is usually accepted that gliomas often progress from low-grade tumors to higher-grade tumors as the time proceeds [12]. The present clinical data and the animal models suggested the immunosurveillance can work in low-grade glioma patients and the immune tolerance would occur in those with high-grade gliomas. The present findings would contribute to the knowledge of molecular basis of low-grade gliomas and the establishment of a novel diagnostic and therapeutic target.

## Materials and methods

### Sera and glioma tissue

Sera were obtained from patients with various types of glioma and from healthy volunteers after they had provided written informed consent. Patients with glioma underwent surgery and the tumor was histologically diagnosed as grade II–IV glioma at Chiba University Hospital in 1998–2008; healthy donors were confirmed to have no cerebral diseases using radiological imaging such as computed tomography or magnetic resonance imaging. No patient received steroid therapy at the time of blood sampling. Each sample was centrifuged at 3 000 × g for 5 min and then frozen at –80°C until use. Glioma tissue was collected from the tumor tissue during surgical treatment. Normal brain tissue, which did not show any glioma cell infiltration under microscopic examination, was isolated from the circumference of the glioma specimen and from non-neoplastic CNS tissues that were obtained during a lesionectomy from a patient with intractable epilepsy or during a lobectomy from patients with benign CNS tumors, such as meningioma. The Local Ethical Review Board of the Graduate School of Medicine, Chiba University approved the studies in this issue, and we obtained written informed consent from the patients and healthy volunteers concerning the use of material for scientific research.

### Phage cDNA library

A total RNA was prepared from the human glioblastoma cell-line U-87 MG (ATCC, HTB-14) using the acid guanidium thiocyanate-phenol-chloroform method with an mRNA purification kit (AquaPure RNA isolation kit, BioRad, Hercules, CA) used in accordance with the manufacturer’s instructions. Double-stranded cDNA was synthesized through conventional procedures and ligated into the EcoRI-XhoI site of λZAP II phage. The library size was over 1.0 × 10^6^ PFU/ml.

### Immunological screening using SEREX

E. coli XL1-Blue MRF’ was infected with λZAP II phages containing a cDNA library and the expression of cDNA was induced by blotting on nitrocellulose membranes, pretreated with 10 mM isopropyl-β-D-thiogalactoside (IPTG; Wako Pure Chemicals, Osaka, Japan). After washing and blocking, the membranes were exposed in 1:2000-diluted serum for 1 h. The membranes were treated with 1:5000-diluted alkalinephosphatase-conjugated goat anti-human IgG (Jackson ImmunoResearch Laboratories, West Grove, PA). After incubation in a color development solution containing 0.3 mg/ml of nitroblue tetrazolium chloride (Wako Pure Chemicals) and 0.15 mg/ml of 5-bromo-4-chloro-3-indolylphosphate (Wako Pure Chemicals), positive reactions were detected. Positive clones were re-cloned twice to obtain monoclonality.

### Sequence analysis of identified clones

Monoclonalized phage cDNA clones were converted to pBluescript phagemids through in vivo excision using ExAssist helper phage (Stratagene, La Jolla, CA). Plasmid DNA was obtained from an E. coli SOLR strain transformed by the phagemid. The inserted cDNAs were sequenced using the dideoxy chain termination method and the sequences were analyzed for homology with a public database provided by the National Center for Biotechnology Information (NCBI).

### Production of glutathione S-transferase (GST) fusion proteins

cDNA inserts of these clones incorporated in pBluescript were cleaved by EcoRI and XhoI generally and cloned into the EcoRI-XhoI site of pGEX-4 T-3, pGEX-4 T-2, and pGEX-4 T-1 vectors (Amersham Bioscience, Piscataway, NJ) that express recombinant GST fusion proteins. E. coli JM109 cells containing pGEX clones (A_600_ = 0.3–0.5) were cultured in 200 ml of Luria broth (LB), and lysed through sonication. The lysate was then centrifuged and the GST-fusion proteins in the supernatants were purified by glutathione-Sepharose. These samples were centrifuged and affinity-purified with glutathione-Sepharose.

### ELISA

Purified recombinant proteins diluted at 10 μg protein/ml in PBS were added to each well of 96-well plates and incubated at room temperature overnight. As a control, the same amount of GST was applied. Sera diluted at 1:100 in PBS with 10% FBS were added to the wells and incubated for 1 h. The wells were exposed to 1:2 000-diluted horseradish peroxidase-conjugated goat anti-human IgG antibody (Jackson ImmunoResearch Laboratories, West Grove, PA). Then, 100 μl of a peroxidase substrate (o-phenylenediamine, 0.4 mg/ml) containing 0.02% (v/v) H_2_O_2_ were added. Absorbance at 490 nm was determined using a microplate reader (Emax, Molecular Devices, Sunnyvale, CA).

### Construction of SH3GL1 deletion mutants

Some deletion constructs of SH3GL1 were obtained through digestion with restriction enzymes or the inverse PCR method. The SEREX-identified phage clone was containing a full-length coding sequence of SH3GL1 (1–368 amino acids), that comprised Bin-Amphiphysin-Rvs (BAR) domain (amino acid positions between 5 and 242) in the N-terminal portion, coiled-coil (CC) domain (amino acid proteins between 180 and 250) at the middle, and the SH3 domain (amino acid positions between 309 and 364) in the C-terminal portion. The region of SH3GL1 cDNA corresponding to amino acids between 260 and 368 was cleaved by SmaI and XhoI and subcloned into the pGEX 4 T-3 vector at the SmaI-XhoI digestion sites (SH3GL1 mut-1). Amino acids 316–368, 260–289 and 354–368 were deleted through the inverse PCR method with the KOD–Plus Mutagenesis Kit (Toyobo, Osaka, Japan) using the SH3GL1 mut-1 cDNA as a template (SH3GL1 mut-2, 3 and 4, respectively). The primers for SH3GL1 mut-2 were forward 5′-CCAGTCTTCCGACAAGCCCATC-3′, reverse 5′-TGGGGATCCACGCGGAACCAG-3′; for SH3GL1 mut-3 were forward 5′-TCGAGCGGCCGCATCGTGAC-3′, reverse 5′-GCCCGACTGGCCGTCCAGCATG-3′; and for SH3GL1 mut-4 were; forward 5′-TCGAGCGGCCGCATCGTGAC-3′, reverse 5′-GCCCGACTGGCCGTCCAGCATG-3′.

### Overlap peptide array

Peptides spanning amino acid residues 1–368 of SH3GL1 were synthesized on cellulose membranes as a series of peptides with the overlapping by 12 amino acids using F-moc amino acids according to the manufacturer’s protocol (Auto spot robot ASP222; ABIMED Analysen-Technik GmbH, Langenfeld, Germany) as previously described [13]. Membranes were incubated with the sera of patients at 1:200 dilutions for more than 12 h. Then, the antigen-antibody complexes were detected with FITC-conjugated goat anti-human IgG (109-095-098; Jackson ImmunoResearch, West Grove, PA) at 1:10000 dilutions. The fluorescence of the peptide spots were detected using Typhoon 9400 (Amersham Biosciences, Stockholm, Sweden) with a 488 nm/520 nm filter. The scanned image was also analyzed with CS analyzer ver. 3.0 (Atto & Rise Corporation, Tokyo, Japan) and fluorescent intensity of each spot was calculated.

### Immunohistochemical staining for SH3GL1 protein

Immunohistochemistry with the polyclonal antibody against SH3GL1 (sc-25495; Santa Cruz) was performed using commercially available reagents, Histofine (Nichirei Bioscience Inc, Tokyo, Japan), and according to the manufacturer’s recommendations. This antibody was confirmed to be cross-reactive for human, mouse, and rat SH3GL1. Sections were counterstained with hematoxylin, then dehydrated and mounted.

Staining of tissue specimens was observed in 100 × fields with approximately all fields presenting glioma cells. The staining intensity in cytosole was classified into 5 groups, absent (−), light partial staining (±), homogeneous light staining (+), partly strong positive staining (++) and homogeneous strong positive staining (+++).

### Brain Tumor Model, Monitoring of Tumor Size, and Serum Sampling

Rat C6 glioma cells and 9 L gliosarcoma cells were originally obtained from ATCC and maintained in Dulbecco's modified Eagle medium (D-MEM) supplemented with 10% fetal calf serum in a humidified atmosphere of 5% CO_2_. Male Wister rats for C6 cells and Fisher rats for 9 L cells, weighing between 200 and 240 g (7–8 weeks old) were used. The animals were anesthetized and placed in a stereotaxic apparatus. A burr hole was made at 4 mm posterior to bregma and 3 mm right to midline. A 25-gauge needle was inserted to the point of 3 mm ventral from dura where 1 × 10^5^ syngeneic C6 or 9 L tumor cells in 10 μl medium were slowly injected. To estimate i.c. tumor volume sequentially, all the animals were examined with a 7 tesla MRI every 7 days started on day 7 after the tumor inoculation. The sera were obtained from tail vein every 7 days. The animal experimentation was reviewed and approved by the Institutional Animal Care and Use Committee of National Institute of Radiological Science.

### Statistical analysis

The significance of differences among healthy donors, patients with low-grade glioma, and patients with high-grade glioma was calculated using the Kruskal Wallis H-test and the Mann–Whitney *U*-test with Bonferroni correction. Differences were considered significant only if p < 0.05. The overall survivals from the date of initial diagnosis were estimated using Kaplan-Meier methodology and compared by the Log rank test to estimate the clinical significance of production of autoantibody for SH3GL1.

## Results

### Serological screening of cDNA library

The phage expression library was constructed using mRNA derived from the U-87 MG glioblastoma cell-line. To identify glioma-associated antigens, a total of 5 × 10^6^ cDNA clones were screened using sera from 48 patients with glioma and 57 reacting clones were isolated from 19 of 48 sera. DNA sequence analysis and a search for homologous sequences in an NCBI-accessible database indicated that these isolated clones comprised 31 independent genes (Table 1).

**Table 1:** Genes identified by SEREX

**Gene name**	**Symbol**	**NCBI accession no.**	**Coding sequence**	**cDNA inserts of recombinant protein**^**†**^
amplified in breast cancer 1	ABC1	NM_022070	18.3563	
anillin, actin binding protein (scraps homolog, Drosophilia)	ANLN	NM_018685	205.3579	
ATP synthase, H + transporting, mitochondrial F1complex, beta polypeptide, nuclear gene encoding mitochondrial protein	ATP5B	NM_001686	106.1695	
catenin (cadherin-associated protein), alpha-like 1	CTNNAL1	NM_003798	22.2248	
CDV3 homolog (mouse)	CDV3	NM_017548	316.1092	
centromere protein F, 350/400 ka (mitosin)	CENPF	NM_016343	175.9519	3553.4866
chromosome 14 open reading frame 145	C14orf145	NM_152446	172.3456	
coagulation factor III (thromboplastin, tissue factor)	F3	NM_001993	124.1011	
coiled-coil domain containing 86	CCDC86	NM_024098	56.1138	
cyclin G1, transcript variant 2	CCNG1	NM_199246	135.1022	
eukaryotic translation elongation factor 1 alpha 1	EEF1A1	NM_001402	64.1452	
ferritin, heavy polypeptide 1	FTH1	NM_002032	236.787	
ferritin, light polypeptide	FTL	NM_000146	200.727	
heterogeneous nuclear ribonucleoprotein C (C1/C2), transcript variant 4	HNRPC	NM_001077443	219.1100	
homeobox B2	HOXB2	NM_002145	121.1191	
Homo sapiens mRNA for KIAA0146 gene, partial cds.	KIAA0146	NM_001080394	1.3218	
macrophage migration inhibitory factor	MIF	NM_002415	98.445	23.561
myosin phosphatase-Rho interacting protein, transcript variant 1	M-RIP	NM_015134	57.3173	2194.3856
nucleolar protein 8	NOL8	NM_017948	304.3807	
oral-facial-digital syndrome 1	OFD1	NM_003611	312.3350	
postmeiotic segregation increased 1(S.cerevisiae)	PMS1	NM_000534	231.3029	
retinoblastoma binding protein 8, transcript variant 1	RBBP8	NM_002894	332.3025	473.1274
ribosomal protein, large, P0, transcript variant 1	RPLP0	NM_001002	179.1131	433.1217
RNA export 1 homolog (S.pombe), transcript variant 1	RAE1	NM_003610	342.1448	
serine/threonine kinase 3(STE20 homolog, yeast)	STK3	NM_006281	142.1617	
SH3-domain GRB2-like 1	SH3GL1	NM_003025	107.1213	43.1615
synaptonemal complex protein SC65	SC65	NM_006455	289.1598	
TAF7 RNA polymerase II, TATA box binding protein (TBP)-associated factor, 55 kDa	TAF7	NM_005642	741.1790	1578.2310
talin 1	TLN1	NM_006289	91.7716	5712…8187
transforming growth factor, beta-induced, 68 kDa	TGFBI	NM_000358	48.2099	1371…2691
unc-45 homolog A (C.elegans), transcript variant 2 or 3	UNC45A	NM_001039675	836.3625	1924.3471

† cDNA inserts of positive clones were successfully expressed into proteins followed by ELISA.

**Figure 1:**
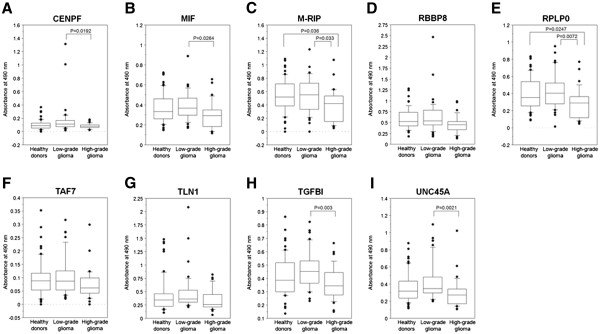
**Serum antibody levels of glioma SEREX antigens. **cDNA inserts of identified clones were recombined in-frame into pGEX vectors that express recombinant GST fusion proteins. Using the fusion proteins as antigens, the levels of antibodies were examined by the ELISA and shown by the ordinate, as **(A)** CENPF, **(B)** MIF, **(C)** M-RIP, **(D)** RBBP8, **(E)** RPLP0, **(F)** TAF7, **(G)** TLN1, **(H)** TGFBI, **(I)** UNC45A. The significance of differences among healthy donors, patients with low-grade glioma and with high-grade glioma was calculated using Kruskal Wallis H-test and Mann–Whitney *U*-test with Bonferroni correction. The box-and-whisker plots display the 10th, 25th, 50th, 75th and 90th percentiles.

**Figure 2:**
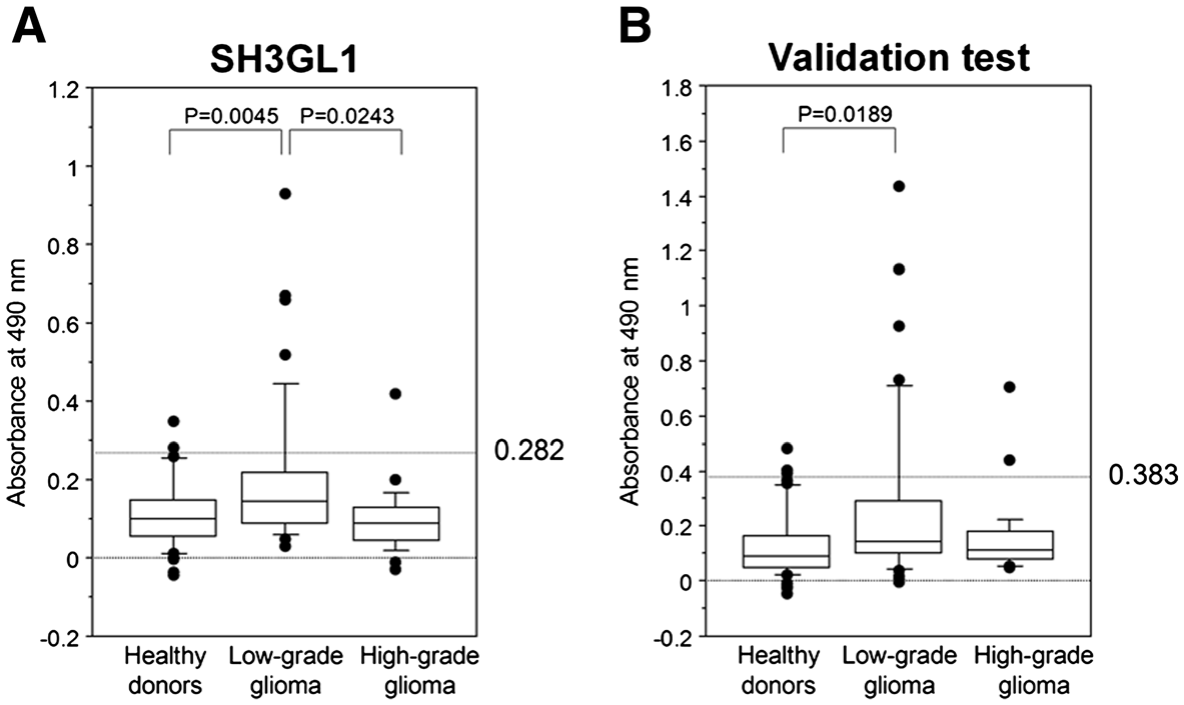
**The increasing levels of antibodies to SH3GL1 in sera of the patients with low-grade glioma.** Serum antibody level to SH3GL1 was examined by the ELISA as described in the legends of Figure 1. First screening test **(A)** and the individual validation test **(B)**, revealed the significant higher levels of autologous antibody against SH3GL1 in low-grade glioma patients, than healthy donors (P = 0.045 and 0.0189).

The GST-fusion recombinant proteins were successfully produced using pGEX-4 T vectors in 10 of 31 antigens—centromere protein F, 350/400 ka (CENPF); macrophage migration inhibitory factor (MIF); myosin phosphatase-Rho interacting protein (M-RIP); retinoblastoma binding protein 8 (RBBP8); ribosomal protein, large, P0 (RPLP0); SH3GL1, TAF7 RNA polymerase II, TATA box binding protein-associated factor, 55 kDa (TAF7); talin 1 (TLN1); transforming growth factor beta-induced 68 kDa (TGFBI), and unc-45 homolog A (UNC45A) (Figures 1 and 2).

### ELISA to detect serum antibodies

Using a recombinant antigen protein, ELISA was performed on sera from 32 patients with high-grade glioma, 40 with low-grade glioma and 56 healthy volunteers, which were collected between 1998 and 2005 in Chiba University Hospital. The serum used for SEREX screening was excluded. The characteristics of the sera are shown in Table 2 (left).

**Table 2:** Characteristics of screening serum sets

	**1st sampling test**	**Validation test**
**Sampling periods**	**1998-2005**	**2005-2008**
**No. of patients**	128	115
**Healthy donors, n (%)**	**56 (43.8)**	**48 (41.7)**
Age (mean ± SD), range	48.1 ± 18.3, 16-76	51.3 ± 15.2, 16-76
**Low-grade glioma, n(%)**	**40 (31.3)**	**42 (36.5)**
Age (mean ± SD), range	45.8 ± 14.8, 20-74	44.2 ± 14.1, 22-78
Pilocytic astrocytoma, n (%)	2 (5.0)	4 (9.5)
Diffuse astrocytoma, n (%)	18 (45.0)	15 (35.7)
Oligodendroglioma, n (%)	16 (40.0)	19 (45.2)
Oligoastrocytoma, n (%)	3 (7.5)	1 (2.4)
Ependymoma, n (%)	1 (2.5)	
Ganglioglioma, n (%)		3 (7.1)
**High-grade glioma, n (%)**	**32 (25.0)**	**25 (21.7)**
Age (mean ± SD), range	49.7 ± 18.3, 8-78	49.8 ± 15.5, 28-78
Glioblastoma, n (%)	24 (75.0)	17 (68.0)
Anaplastic astrocytoma, n (%)	5 (15.6)	3 (12.0)
Anaplastic oligodendroglioma, n (%)	2 (6.3)	2 (8.0)
Anaplastic oligoastrocytoma, n (%)	1 (3.1)	1 (4.0)
Anaplastic ependymoma, n (%)		1 (4.0)
Choroid plexus carcinoma, n (%)		1 (4.0)

The levels of serum antibodies of CENPF, MIF, M-RIP, RPLP0, TGFBI and UNC45A were significantly lower in patients with high-grade glioma than in those with low-grade glioma (Figure 1A-C, E, H and I) and, moreover, the levels of anti-M-RIP and anti-RPLP0 antibodies in patients with high-grade glioma were also significantly lower than in healthy volunteers (Figure 1C and E).

The levels of serum antibodies to SH3GL1 were significantly higher in patients with low-grade glioma than those with high-grade glioma (P = 0.0243) and healthy volunteers (P = 0.0045) (Figure 2A). When the antibody levels were divided into 2 groups with a cut-off value of 0.383 corresponding to the mean + 2 standard deviations (SD) of SH3GL1 antibodies in healthy volunteers, the positive rate of patients with low-grade glioma was 62.5% (25 of 40), whereas those of patients with high-grade glioma and healthy volunteers were 8.9% (5 of 56) and 15.6% (5 of 32), respectively.

### Independent validation test for the levels of antibodies to SH3GL1

To verify the generality of low-grade glioma-specific increase in serum antibodies to SH3GL1, an independent validation test was carried out using other serum set. In validations, consecutive serum samples that were collected in 2005–2008 after the first serum sampling, were enrolled, and no apparent differences in the characteristics were observed between the 2 serum sets (Table 2). The results of the ELISA based on the newly collected serum set showed that the levels of serum autoantibodies to SH3GL1 were significantly higher than those of healthy donors (P = 0.0189) (Figure 2B). Although there was no statistical significance in the levels of antigens between patients with low- and high-grade glioma, similar distribution was recognized. In the combined population of the first sampling test and the validation test, there was a significant difference between low-grade gliomas and high-grade gliomas (p = 0.0351).

The same results of both ELISA based on the independent serum sets support the possibility that SH3GL1 is aberrantly expressed and efficiently elicits a systemic immune response in low-grade glioma patients. The level of anti-SH3GL1 autoantibody could be a novel low-grade glioma-specific serum marker. In contrast, the lower serum autoantibody levels against these determined SEREX-antigens in patients with high-grade glioma as opposed to those with low-grade glioma and healthy volunteers suggest that the existence of some immunosuppressive mechanisms in high-grade gliomas.

### Patients survival

**Figure 3:**
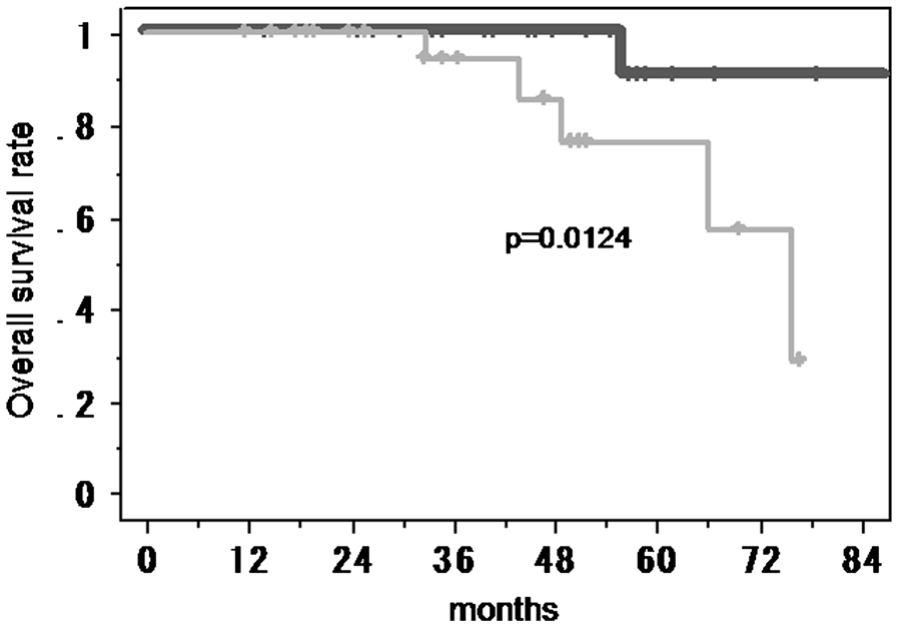
**Kaplan-Meier analysis for the overall survival of the patients with low-grade gliomas according to the serum level of anti-SH3GL1 autoantibody.** The patients with higher serum level of anti-SH3GL1 autoantibody (solid line) survived significantly longer than those with lower levels (gray line) (p = 0.0124).

Overall survival of the patients with low-grade gliomas according to the serum level of anti-SH3GL1 autoantibody was analyzed by Kaplan-Meier analysis. The patients included in the test set and the validation set were divided into 2 groups with a cut-off value of the mean + 1 SD of anti-SH3GL1 antibodies in healthy volunteers. The patients with higher serum level of anti-SH3GL1 autoantibody survived significantly longer than those with lower levels (p = 0.0124) (Figure 3).

### Search for epitope sites of SH3GL1

**Figure 4:**
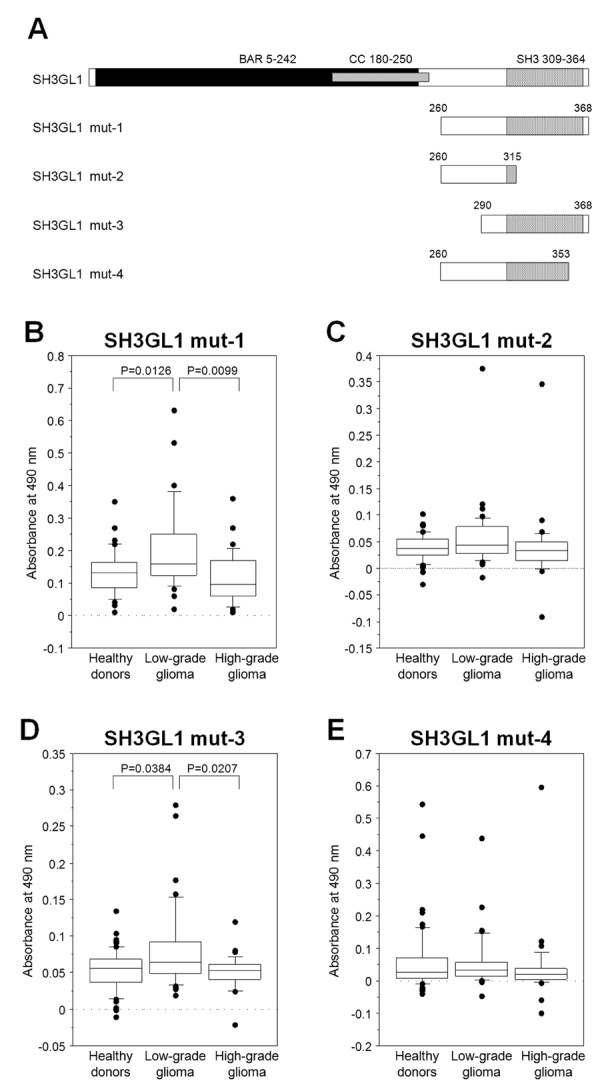
**Comparison of serum antibody levels among deletion mutants of SH3GL1.** To confirm the epitope site, some SH3GL1 deletion mutants **(A)** were synthesized. Serum antibody levels were examined by ELISA with SH3GL1 muta-1 **(B)**, mut-2 **(C)**, mut-3 **(D)** and mut-4 **(E)**, and the 10–20 amino acids at the C-terminal end were indicated as the epitope site.

To determine the accurate immuno-reactive site, an ELISA using 4 deletion mutants of SH3GL1 cDNA was performed. The BAR domain deletion mutant, identified as SH3GL1 mut-1, was obtained first, and the N-terminal and C-terminal deletion mutants of SH3GL1 mut-1 were produced, as SH3 mut-2 and 3, respectively (Figure 4A). The serum antibody levels to SH3GL1 mut-1 and mut-3 in the patients with low-grade glioma were still significantly higher than those in other groups (Figures 4B and D), while the levels of anti-SH3GL1 mut-2 showed no difference among the groups (Figure 4C). Although these results indicated that the C-terminal of SH3GL1 contributed to the immune-response, the differences were disappeared in SH3GL1 mut-4, deleting only 15 amino acids at the 3′ end of SH3GL1 mut-1 (Figure 4D). These results were suitable for that of overlap peptide array, and approximately the 15 amino acids in the C-terminal of SH3GL1 are indispensable as the epitope recognized by serum antibodies in the patient with low-grade glioma.

**Figure 5:**
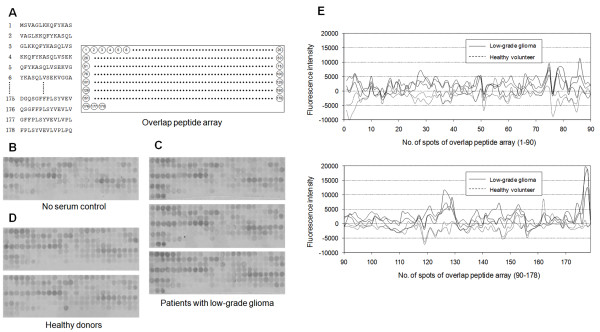
**The detection of epitope site by overlapped peptide array.** Series of peptides of 14 amino acid residues, composed of SH3GL1, were synthesized with overlapping by 12 amino acids, and were blotted in nitrocellulose membranes using F-moc amino acids **(A)**. Three sera of the patients with low-grade glioma indicated the fine reaction in spot 177 and 178 **(C)**, compared to two normal volunteers **(D)** and no serum control **(B)**. The calculated fluorescence intensity, normalized by background control, revealed that these spots were suggested as a minimum epitope site **(E).**

To confirm the epitope site in the deletion mutant ELISA, overlap peptide array, which is a much useful analysis based on the SPOT-synthesis technique, was applied. In the present analysis, series of peptides of 14 amino acid residues, composed of full-length SH3GL1, were synthesized with overlapping by 12 amino acids, and were blotted in nitrocellulose membranes using F-moc amino acids (Figure 5A). Three representative higher immune-reactive sera of the patients with low-grade glioma, two of the normal volunteers and PBS without serum as background control, were applied in the peptide array (Figure 5B-C). All of three sera of patients showed the fine specific reaction in two consecutive blots, spot 177 and 178, indicating the C-terminal-end of SH3GL1, comparing with the sera from normal volunteers. The calculated fluorescence intensity normalized by background control (Figure 5E) revealed that the common sequence in 2 reactive blots, FPLSYVEVLVPL, was suggested as a minimum epitope site.

### Immunohistochemical staining for SH3GL1 protein

**Figure 6:**
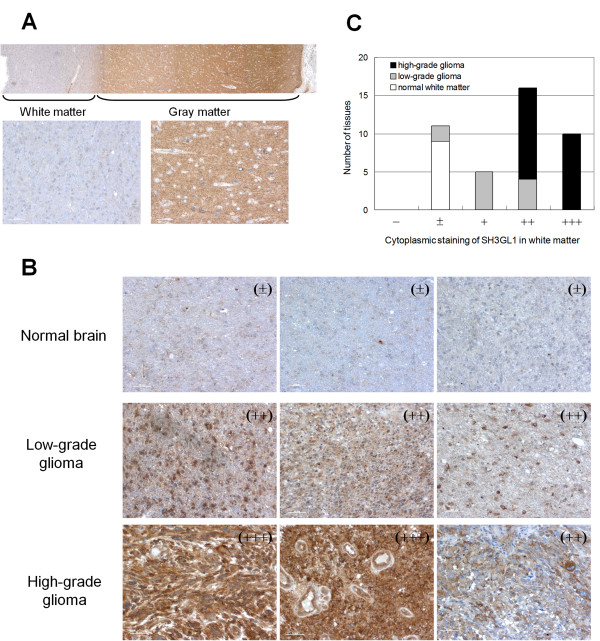
**Immunohistochemical analysis of SH3GL1 in glioma cells.** Immunohistochemical stain for SH3GL1 in whole normal brain, consisted of white matter and gray matter **(A)**, and three representative results of normal white matter, low-grade glioma and high-grade glioma **(B)** were shown. Immunostaining for SH3GL1 was classified in five groups, and numbers of tissues in each group were scored **(C).**

To verify the SH3GL1 expression in glioma tissues directly, immunohistochemical stains for SH3GL1 was obtained in normal brain, low-grade glioma and high-grade glioma. In the normal brain, clear contrast was observed between gray matter (cerebral cortex) and white matter (medulla) (Figure 6A). In the gray matter, where neuronal cells (neurons) abundantly existed, cytoplasm was stained homogeneously, while nuclei were occasionally stained in white matter, which contained mainly glial cells.

It is known that glioma cells are commonly localized in white matter and progress along neural fibers [14]. Therefore, we compare the immunostaining levels between normal glial cells in white matter and glioma cells. In glioma tissues, strong positive staining of SH3GL1 was observed in the cytoplasms but not in the nucleus (Figure 6B). The levels of stain in white matter increased according to the malignancy of tumors; that is, high-grade glioma tissues were most heavily stained while normal glial cells were barely stained (Figures 6C). These results indicated that the protein levels of SH3GL1 were much higher in glioma cells than in normal glial cells in white matter.

### Alteration of anti-SH3GL1 autoantibody level in rat glioma model

**Figure 7:**
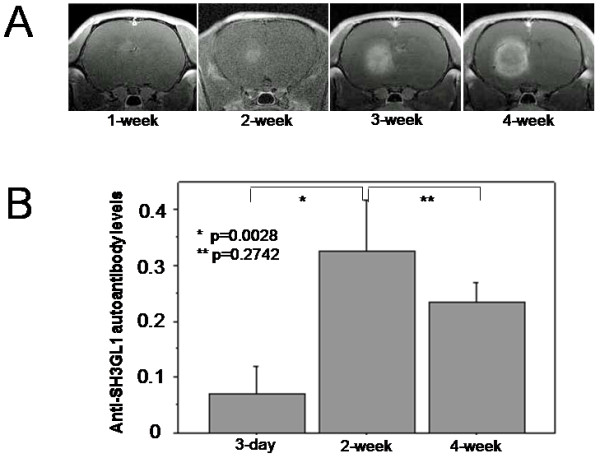
**Changes in the serum autoantibody level to SH3GL1 in a rat brain tumor model using C6 rat glioblastoma cells which were confirmed to express SH3GL1 protein.** MRI studies show a steady growth of tumor mass in the rat brain **(A)**. The serum autoantibody levels were significantly increased at 2-week after tumor inoculation, and tended to decrease at 4-week after the inoculation **(B).**

To confirm the changes in the serum anti-SH3GL1 autoantibody level, we used rat glioma models with C6 and 9 L cells which expressed its messenger RNA (data not shown). In the models, the brain tumors constantly became visible on MRI at 2-week after tumor inoculation and over 200 mm^3^ at 4-week (Figure 7A). All the tumor-bearing animals died within 5 weeks from the tumor inoculation. In the C6 glioma model, the serum levels of autoantibody to SH3GL1 significantly increased in the rats at 2-week after tumor inoculation compared with those at 3-day after the inoculation (p = 0.0028) (Figure 7B). In contrast, at the time of 4-week after the inoculation, the serum levels tended to decrease. In the other experiment using 9 L gliosarcoma cells, the result showed the same tendency without statistical significance (data not shown). These results show that the serum levels of autoantibody to SH3GL1 increased at the early stage of the animal models and turned to decrease at the late stage according to the increase of tumor volume as the time proceeded.

## Discussion

The molecular pathogenesis of glioblastoma has been well characterized and involves both gain and loss of a number of genes participating in proliferative or mitogenic signals. One of the most prevalent molecular changes consists of aberrant activation of EGFR, which occur in 50% of glioblastoma, but not seen in low-grade astrocytomas [12,15]. We have shown in this study that the SH3-domain of GRB2-like protein, which links the receptor tyrosine kinases activation to the ras pathway, had already overexpressed in low-grade gliomas and strongly induced a humoral immune response. In high-grade gliomas, the tissue expression of SH3GL1 was further increased, but the immune response was suppressed. Although there are few reports describing overexpression of this protein in human cancers, SH3GL1 protein is related to the activation of MLL proto-oncogene by chromosomal translocation [16]. Solitary SH3GL1 overexpression in NIH3T3 cells also reported to do some oncogenic behaviors in vivo [17,18]. It is not clear whether the overexpression is a result of amplification of receptor tyrosine kinases or not. However, the net result of these signaling complexes induces the shift of ras-GDP to its activated form ras-GTP, and may lead to activate the MAPK cascade and resultant alteration in gene expression concerning cell proliferation.

SH3GL1 is known to be predominantly localized in the nuclei of haematopoietic cells and fibroblasts in contrast to cytoplasmic localization in neurons and osteoblasts [19,20]. In the adult brain, SH3GL1 is highest in the neurons of the granular layer of the cerebral cortex [21] and known to be involved in the development of central nervous system [22,23]. These published data were compatible with our results of immunohistochemical staining with SH3GL1 antibody. In glioma tissues, strong positive staining of SH3GL1 was obtained in the cytoplasms but not in the nucleus, and the levels of staining in white matter increased according to the advance of its malignancy. These results suggested that the SH3GL1 overexpression might have some oncogenic roles in gliomas. However, the levels of serum autoantibodies to SH3GL1 in the patients with high-grade glioma were not increased in our study, while the levels in the patients with low-grade glioma were increased. It is believed that the abnormal cytoplasmic SH3GL1 overexpression in glioma cell has a potential to induce immune responses, but various mechanisms of immunosuppression prevent the reaction in high-grade glioma [24-27]. All the other candidate genes identified in this study showed the same low immunoreactivity in patients with high-grade gliomas. The suppression of the immunosurveillance mechanism in high-grade glioma would attenuate the recognition of SEREX-derived antigens in antigen presenting cells (APC). In fact, it has been known that various immunosuppressive molecules, such as TGF-β, IL-10, and prostaglandins, are highly expressed in cancers including high-grade glioma [24,25], and these molecules could inhibit the maturation of professional APCs. Such an evading immune destruction has now added to the hallmark of cancer [28].

The major cause of the lower level of anti-SH3GL1 autoantibody in high-grade glioma patients would be the non-specific immunosuppression caused by increased immunosuppressive cytokines [24,25]. However, the animal experiment provides an additional hypothesis that the depressed autoantibody levels could be partly due to the antigen-specific immune tolerance induced by the existence of large tumor and long-term antigen exposure. The early stage of the rat glioma models indicates a relatively small tumor and short-term antigen exposure, and the late stage indicates a large tumor and long-term antigen exposure to the immune system. The long-term antigen exposure from a large tumor could generally induce immune tolerance through development of immune resistant tumor variants and the tumor microenvironment inducing immune cell anergy or death [26,27]. It is usually accepted that gliomas often progress from low-grade tumors to higher-grade tumors as the time proceeds, although low-grade gliomas are not always in an early-stage of the disease and secondary glioblastoma is less frequent than de novo glioblastoma [12]. The possible contribution of antigen-specific immune tolerance to the depressed autoantibody levels in high-grade glioma patients remains to be elucidated.

SEREX is one of the most powerful tools to find novel tumor antigen for various cancers [5], and some autologous antigens to esophageal cancer have been identified in our groups [29-31]. Compared with other screening techniques such as transcriptomics or proteomics, SEREX offers a crucial advantage that subtle changes in the protein expressions can be detected through immunological reactions [32,33]. Several authors have already applied SEREX to glioma, and some antigens, including glioma-expressed antigen 2 (GLEA2) [7], PHD finger protein 3 (PHF3) [7,34], and SRY-box 6 (SOX6) [8] have been identified. It should be noted that we found autologous antibodies against SH3GL1 to be a low-grade glioma-specific marker with similar experimental systems to others. Our unique approach was the quantitative comparison of the levels of serum antibodies using the ELISA, while the approach of others was qualitative analysis. The application of ELISA in the validation step could lead to the discovery of a low-grade glioma-specific high titer of the autoantibody and the decrease in high-grade gliomas. Although some candidates of glioma biomarkers have been identified by various screening methods [6-8,34-37], no serum marker for early diagnosis has been found yet. Therefore, it is quite valuable to find a novel serum biomarker for its early diagnosis, prediction of the prognosis in each patient, and development of a new molecular target. Indeed,

The results of an overlap peptide array and ELISA using deletion mutants of SH3GL1 showed that 12 amino acids in the C-terminal portion, FPLSYVEVLVPL, were indicated as a major epitope site. By using a synthetic peptide corresponding to the epitope as an antigen, a more accurate screening for the patients with low-grade gliomas and a specific peptide vaccine therapy would be achieved in the future.

## Competing interests

The authors declare that they have no competing interests.

## Authors’ contributions

TM performed experiments, analyzed data and participated in writing; TH, MT, NS, and YI conceived the idea, designed and supervised the study; TO carried out immunohistochemistry; MK performed the overlap peptide array. All authors read and approved the final manuscript.

## Author details

^1^Departments of Neurological Surgery, Chiba University, Graduate School of Medicine, 1-8-1, Inohana, Chuo-ku, Chiba 260-8670, Japan. ^2^Genetics and Biochemistry, Chiba University, Graduate School of Medicine, 1-8-1, Inohana, Chuo-ku, Chiba 260-8670, Japan. ^3^Diagnostic Pathology, Chiba University, Graduate School of Medicine, 1-8-1, Inohana, Chuo-ku, Chiba 260-8670, Japan. ^4^Department of Biochemistry, Graduate School of Life Science, Nagoya Women’s University, 3-40, Shioji-cho, Mizuho-ku, Nagoya 467-8610, Japan.
